# Nurse‐sensitive quality and benchmarking in hospitals striving for Magnet® or Pathway® designation: A qualitative study

**DOI:** 10.1111/jan.16245

**Published:** 2024-05-27

**Authors:** Claudia B. Maier, Carolin Gurisch, Julia Köppen, Joan Kleine, Linda H. Aiken

**Affiliations:** ^1^ School of Public Health Bielefeld University Bielefeld Germany; ^2^ Department of Health Sciences BQS Institut für Qualität Und Patientensicherheit GmbH Hamburg Germany; ^3^ Department of Healthcare Management Technische Universität Berlin Berlin Germany; ^4^ School of Nursing, Center for Health Outcomes and Policy Research University of Pennsylvania Philadelphia USA

**Keywords:** benchmarking, hospital, indicators, Magnet®, nurse, Pathway®, qualitative analysis

## Abstract

**Aim:**

To examine if and how selected German hospitals use nurse‐sensitive clinical indicators and perspectives on national/international benchmarking.

**Design:**

Qualitative study.

**Methods:**

In 2020, 18 expert interviews were conducted with key informants from five purposively selected hospitals, being the first in Germany implementing Magnet® or Pathway®. Interviews were analyzed using content analysis with deductive‐inductive coding. The study followed the COREQ guideline.

**Results:**

Three major themes emerged: first, limited pre‐existence of and necessity for nurse‐sensitive data. Although most interviewees reported data collection for hospital‐acquired pressure ulcers and falls with injuries, implementation varied and interviewees highlighted the necessity to develop additional nurse‐sensitive indicators for the German context. Second, the theme creating an enabling data environment comprised building clinicians' acceptance, establishing a data culture, and reducing workload by using electronic health records. Third, challenges and opportunities in establishing benchmarking were identified but most interviewees called for a national or European benchmarking system.

**Conclusion:**

The need for further development of nurse‐sensitive clinical indicators and its implementation in practice was highlighted. Several actions were suggested at hospital level to establish an enabling data environment in clinical care, including a nationwide or European benchmarking system.

**Implications for the Profession and Patient Care:**

Involving nurses in data collection, comparison and benchmarking of nurse‐sensitive indicators and their use in practice can improve quality of patient care.

**Impact:**

Nurse‐sensitive indicators were rarely collected, and a need for action was identified. The study results show research needs on nurse‐sensitive indicators for Germany and Europe. Measures were identified to create an enabling data environment in hospitals. An initiative was started in Germany to establish a nurse‐sensitive benchmarking capacity.

**Patient or Public Contribution:**

Clinical practitioners and nurse/clinical managers were interviewed.

## 1 INTRODUCTION

The quality of hospital care and patient safety is an issue of concern globally (Kohn et al., 2000). Across and within countries, health system and hospital performance is highly variable (Busse et al., 2019; OECD, 2022). A multitude of quality improvement strategies and initiatives have been implemented worldwide (Busse et al., 2019; Slawomirski et al., 2017; World Health Organization, 2018). Examples include hospital quality management and accreditation schemes, regulations, clinical audits, practice guidelines, team‐based quality improvement cycles, as well as public reporting (ibid).

Nurses represent the largest profession providing direct patient care in hospitals and other healthcare settings in most countries worldwide (World Health Organization, 2020). Hence, measuring nurses' contribution to quality of care has received increased attention (Afaneh et al., 2021). There are several definitions of nurse‐sensitive quality (American Nurses Association, 2023; Mueller & Karon, 2004; Oner et al., 2021). Definitions and use of nurse‐sensitive indicators vary by country and setting, e.g., hospital, ambulatory or long‐term care (ibid). Yet, common elements of the definitions are the direct and measurable contribution of nurses associated with outcomes (Afaneh et al., 2021; Mushta et al., 2018; Oner et al., 2021). One review defined nurse‐sensitive indicators as ‘changes in a person's health status that nursing care can directly affect’ (Heslop et al., 2014).

### 1.1 Background

Internationally, one initiative to improve and measure nurses' contribution to quality of care is the Magnet®‐model of organizational redesign which aims to improve hospital work environments and nursing excellence in clinical care and patient outcomes (American Nurses Credentialing Center, 2021). The Magnet®‐model comprises five components: transformational leadership, structural empowerment, exemplary professional practice, new knowledge, innovation and improvements, and empirical outcomes (American Nurses Credentialing Center, 2021). As of March 2024, a total of 591 organizations have received official Magnet®‐designation. The majority are hospitals in the United States (U.S.), 17 hospitals exist globally of which only one is located in Europe (American Nurses Credentialing Center, 2023). Across Europe, the EU‐funded Magnet4Europe study, which has started in 2020, implements Magnet® in over 60 hospitals (Belgium, England, Germany, Ireland, Norway and Sweden). It uses a randomized controlled trial and a qualitative process evaluation (Sermeus et al., 2022). The intervention comprises one‐to‐one twinning of each European hospital with a U.S. Magnet® hospital, gap analyses to document features of the current practice environment, use of the Magnet® blueprint, combined with international learning collaboratives, national networks and yearly monitoring and feed‐back reports (Reich et al., 2022; Sermeus et al., 2022). Prior to Magnet4Europe, a feasibility study (Magnet‐Pioneer) was conducted in Germany to identify hospitals which had started implementing Magnet® or Pathway® prior to the Magnet4Europe study, hence to identify pioneers or early adopter hospitals.

One of the five central components of Magnet® is empirical outcomes, including nurse‐sensitive data and benchmarking. Magnet® designation requires hospitals to collect and benchmark nurse‐sensitive clinical indicators (American Nurses Credentialing Center, 2023). For inpatient units, two indicators are mandatory and pre‐defined, these are hospital acquired pressure ulcers and falls with injury. In addition, two indicators have to be selected from a list of inpatient indicators, plus additional indicators for ambulatory care (American Nurses Credentialing Center, 2021).

Most research on nurse‐ sensitive data and outcomes has focused on North America. In the U.S., a set of indicators was developed by the American Nurses Association (ANA) and the U.S. Agency for Healthcare Research and Quality, and are included in the National Database for Nursing Quality Improvement (NDNQI), which many hospitals use as part of their Magnet journey (American Nurses Association, 2023; Press Ganey, 2023). In Canada, an initiative was started to develop standardized quality measures reflective of nursing care, the Canadian Health Outcomes for Better Information and Care (C‐HOBIC) project (Canadian Nurses Association & Canadian Institute for Health Information, 2023; Hannah et al., 2012). In other countries, including in Germany, there has been limited research on nurse‐sensitive data or benchmarking.

In Germany, despite several legal requirements for hospitals to measure, monitor and report quality (§ 135a Social Law Book (SGB) V), there has been limited focus on the contribution of nurses to quality of care. Nurse‐sensitive indicators have been described in Germany as parameters with a correlation between the number of nursing staff and the incidence of adverse events (National Association of Statutory Health Insurance Funds & German Hospital Federation, 2018; Schreyögg & Milstein, 2016), but there is no nationwide, standardized data collection which includes nurse‐sensitive indicators, except for pressure ulcers which is routinely collected by the Institute for Quality Assurance and Transparency in Healthcare (Institute for quality assurance and transparency in healthcare, 2023). Yet, the data have been criticized for their limited validity, inadequate risk adjustment and the long time lag between data collection and reporting (Geraedts & Cruppé, 2022; Maass et al., 2011; Vorbeck et al., 2022). In addition, German hospitals are by law (§ 108 SGB V) required to publish annual quality reports online to increase self‐reporting as well as public attention, however, apart from data on pressure ulcers, this does not include information on nurse‐sensitive indicators (Federal Joint Committee, 2022).

The German Federal Ministry of Health funded 10 projects from 2003 to 2007 as part of the funding program ´Benchmarking in patient care`. The projects pursued the goal to improve patient care by participating in benchmarking and covered heterogeneous topics, for instance, stroke and geriatric care (Zylka‐Menhorn & Gerst, 2007). Yet, the benchmarking projects did not focus on developing and comparing nurse‐sensitive quality indicators.

### 1.2 The study

Against the backdrop of limited research on nurse‐sensitive data and implementation in hospitals in Germany, the aim of this explorative study was to explore in a small number of selected hospitals the existence and use of nurse‐sensitive quality indicators, implementation and views on national and international benchmarking from the perspective of hospital managers, clinical leaders and other key resource persons.

## 2 METHOD

### 2.1 Study design

A qualitative study was conducted as part of a feasibility study (Magnet‐Pioneer). Magnet‐Pioneer was designed as explorative study, which identified German pioneer hospitals known as having begun implementing Magnet® or Pathway® at their own initiative prior to 2019/2020, hence prior to the EU‐Magnet4Europe study. The aim of the feasibility study was therefore to identify and gain expert views from these pioneer hospitals in Germany, the reasons for implementing Magnet®, the feasibility and views on nurse‐sensitive data and benchmarking.

The study followed the consolidated criteria for reporting qualitative research (COREQ) (Tong et al., 2007) (Appendix S1).

### 2.2 Study setting and recruitment

Five eligible hospitals were selected in 2019/2020. Hospital inclusion criteria comprised hospitals (i) in the process of implementing Magnet® or Pathway® and (ii) being among the first hospitals in Germany in the implementation process. Pathway® shares similarities with Magnet® insofar as it also fosters and recognizes excellent practice environments, for instance by empowering nursing staff and supportive leadership. It focuses on six standards of practice and there are fewer data requirements and no requirement for benchmarking compared to Magnet®.

The sampling was purposive, individual interviewees were chosen if the following criteria applied: (i) working as chief nursing officer (CNO) or in another leading role at the management level and having experience with the implementation of Magnet®/Pathway®, (ii) or having a key role in implementing Magnet®/Pathway® in clinical care, e. g. as a nurse specialist or physician. In addition, the snowball method was used to identify suitable interview partners in the hospitals. In each hospital persons of contact were informed about the planned interviews and asked to forward the request for an interview to those fulfilling the predefined criteria. After suitable interview partners had been named, they were sent an invitation letter for the interview via e‐mail. All requested interview partners agreed to be interviewed. All interviews took place once, there were no repeat interviews.

### 2.3 Data collection

In total, 18 expert interviews were conducted with chief nursing officers, physicians, managers and nursing staff of the selected five hospitals. The interviews were conducted on‐site at the interviewees' workplaces with the exception of one interview which was conducted at the interviewer's workplace. All interviews were conducted over a period of eight months in 2020 and carried out by three researchers, after a test phase to ensure consistency in interviewing. Apart from the researcher and the interviewee, no other persons were present during the interviews. All interviews lasted between 30 and 135 min and were recorded by a voice recorder, after informed consent was given and consent forms were filled in. Sampling was stopped after data saturation was reached and no new topics were identified (Glaser & Strauss, 1967).

A semi‐structured guide was developed and piloted, consisting of three main themes, first the rationale and motivation for implementing Magnet®/Pathway®, second, the experiences with implementation (covering the five Magnet® components: leadership, empowerment, clinical practice, new knowledge/innovation, and empirical data/outcomes) and third, lessons with regard to the transferability of a U.S. concept to Germany, including data and benchmarking. As part of the themes ‘empirical data/outcomes’ as well as ‘transferability of a U.S. concept to Germany' interviewees were asked about nurse‐sensitive data and benchmarking, which is a central component of Magnet.

In addition to the interviews, all interviewees were asked to fill out a short questionnaire, which included job titles, years of work experience, educational background (nursing, medicine, other) demographics, and questions about their previous knowledge of and experience with Magnet®/Pathway®.

### 2.4 Data analysis

The recorded interviews were transcribed verbatim. The transcripts were not sent back to the interviewees. Interviewees' names or other potentially identifiable information were removed, each interviewee received a pseudonym and potentially identifiable quotes were anonymized. Coding was performed with Atlas.ti based on qualitative content analysis (Mayring, 2021) and a deductive‐inductive approach. This approach was chosen to identify the relevant interview material on nurse‐sensitive data and benchmarking. Coding was divided into two steps.

First, codes were formed deductively following the main questions of the interview guidelines. Therefore a codebook was developed. For this study, the interview material specifically on data, outcomes and benchmarking was identified and coded. It covered interviewees' views and experiences with the existence of nurse‐sensitive data in the hospitals, related challenges, use of indicators, implementation process as well as their views on benchmarking opportunities and barriers in the German healthcare context. Coding was performed by three researchers.

Second, the material on data, empirical outcomes and benchmarking was coded inductively (Mayring, 2021). The text was re‐read and paraphrased by a fourth researcher with the aim of a more precise understanding of the focused topic. In an iterative process, including repeated exchanges between all four researchers, codes were formed inductively and summarized in three main themes and nine subthemes. The results use both, inductive coding for sub‐themes within the overall deductive themes of data, empirical outcomes and benchmarking. All codes were noted in a codebook and meaningful quotes translated into English by the authors (Appendix S2). Codes and quotes were translated after data analysis. To ensure the quality and accuracy of the translation, this step was carried out by two researchers.

### 2.5 Ethical considerations

Ethics approval was obtained January 9th, 2020 by the ethics committee of the Charité (No. EA4/185/19). In advance to the interview as well as on the day of the interview, interviewees were repeatedly informed about the study's objective and background, the voluntariness of participation and data protection. On the day of the interview the interviewers presented themselves, explained their position at the university and their role in the research; and informed consent in writing was provided by all interviewees. Consent could have been withdrawn at any time without consequences for the interviewees, but no one had made use of this option. The results and illustrative quotes were anonymized, any references to the identity of the interviewees were removed from the data analysis and the quotes. Interviewees were assured confidentiality.

### 2.6 Rigor and reflexivity

Recommendations for quality assurance in the conduct of qualitative studies were followed (Glaser & Strauss, 1967). To ensure rigor and reflexivity, the focus was set on researcher triangulation. Other forms of triangulation were not undertaken (e.g. no data triangulation of different types of data, no theory triangulation or methodology triangulation). Interviews were conducted after prior training and piloting of interview techniques of interviewers to ensure comparability of interviewing. Several rounds of pilots and discussions were conducted among the researchers to ensure consistency in the coding process. Research findings and interpretation were discussed within the research team. In an iterative process, coded data was summarized in main themes and sub‐themes after consent of the research team was reached. A thorough description of the research method and the provision of interview quotes and associated codes support this research's dependability and transferability.

## 3 RESULTS

### 3.1 Sample description

Of the 18 interviewees, eleven were male and seven female. All but one had leadership and/or staff responsibility. A total of 16 had a qualification background in nursing, two in medicine. Interviewees were on average 48.9 years old (range 33–67 years) and had 27.8 years (range 11–40 years) professional experience in healthcare. The self‐reported knowledge of Magnet®/Pathway® ranged between one and 15 years, the experience with implementation between one and twelve years. The number of beds in the hospitals was between 200 and 2000 beds.

### 3.2 Major themes and subthemes

Three major themes emerged from the interview coding: first, limited pre‐existence of and necessity for nurse‐sensitive data, second, creating an enabling data environment, and third, challenges and opportunities in establishing benchmarking. Table 1 provides an overview of the major themes and related subthemes.

**TABLE 1:** Overview of major themes and subthemes from 18 interviews.

Major themes	Subthemes	Description
Limited pre‐existence of and necessity for nurse‐sensitive data	Pre‐existing nurse‐sensitive indicators and data collection	Data collection of pressure ulcers, falls with injuries and other nurse‐sensitive data, need for developing further nurse‐sensitive data
	Inhouse‐development of nurse‐sensitive data collection	Hospital‐specific selection, development of nurse‐sensitive data collection
Creating an enabling data environment	Creating clinician acceptance for data collection	Generating motivation for data collection/usage, academic degree as facilitating factor
	Establishing a data culture	Data approach, data leadership, data visualization, involvement of clinicians in content‐related topics for data collection and illustration, building data literacy
	Usage of electronic health records	Status of implementation, personnel and financial efforts, benefits
Challenges and opportunities in establishing benchmarking	Implementation of internal benchmarking	Benchmarking within a hospital, within hospital groups
	Limited possibilities for national and international benchmarking	Nationwide benchmarking, benchmarking across sub‐groups of hospitals, benchmarking within Europe, benchmarking within the U.S.
	Motivation for and challenges of setting up benchmarking	Benefits: Improvement of patient care, improved visibility of nursing performance, fostering nursing research challenges: identifying comparison groups, ensuring data standardization, ensuring data quality

#### 3.2.1 Limited pre‐existence of and necessity for nurse‐sensitive data

Most interviewees reported a general lack or paucity of existing nurse‐sensitive data in their hospitals. At the same time, they highlighted the necessity for having good‐quality indicators available. This dual‐message came across frequently, hence was formed as one major theme. The first sub‐theme focuses on the pre‐existence as well as limitations of nurse‐sensitive data, whereas the second sub‐theme focuses on the developments of nurse‐sensitive data that individual hospitals or key resource persons were involved in.

##### Pre‐existing nurse‐sensitive indicators and data collection

Most respondents reported to collect data or are in the process of implementing data collection on falls with injuries and pressure ulcers (12 out of 18 respondents):

‘(…) falls with injury, pressure ulcer, much of this has already been measured.’(Interviewee 1)

‘What we've already taken on, for example, are these data collections on falls and pressure ulcers for the individual wards.’ (Interviewee 6)

Data collection for pressure ulcers is legally required for German hospitals, making this data commonly available, which was mentioned by several interviewees.

The implementation status of collecting other nurse‐sensitive indicators varied considerably. It ranged from the decision to select nurse‐sensitive quality indicators for data collection, implementation of data collection, to sporadically implemented data collection in practice (9 out of 18 respondents). Most statements made clear that the implementation of further nurse‐sensitive data collection was at the beginning, and simultaneously underlined the need for collecting nurse‐sensitive data:

‘Then we said, what is important for us? And then we said, central venous line infection. (…) we need central venous lines in practice. (…). They weren't really captured at all, just imagine.’ (Interviewee 3)

‘Other areas are starting to collect data for urinary tract infections. We don't have that in my area. (…) We are dealing with the indicators for falls and pressure ulcers. That's what we're working on.’(Interviewee 5)

For the choice of these indicators, practicability in terms of implementation, sufficient staff, and relevance for clinical practice were the highlighted aspects underpinning discussions on indicator selection. Interviewees reported to collect data or to establish data collection for infection rates (catheter associated urinary tract infections, infection rates induced by central venous catheters, MRSA [methicillin‐resistant staphylococcus aureus] infection rates), the time between hospital arrival and placing the injection needle when treating stroke patients (door‐to‐needle time) and measures taken to restrict physical movement of patients (patient fixation).

##### Inhouse‐development of nurse‐sensitive data collection

A small number of interviewees mentioned that they were developing their own nurse‐sensitive indicators (4 out of 18 respondents), for instance on quality of life, independence and consequences of falls:

‘What is complimentary in our case, (…) is the data collection of quality of life and autonomy indicators. (…). We want to reach a completely different level than is actually typical in the US. (…) Because a patient does not come to the hospital in order not to get a hospital acquired pressure ulcer or not to get an infection, but to be independent again afterwards, as autonomous as possible. That's what nursing is all about, and we wanted to record that in a questionnaire so that the difference between good and not‐so‐good nursing care can be measured.’ (Interviewee 1)

Reasons for the development of these indicators include the relevance for and possibility to improve patient care.

#### 3.2.2 Creating an enabling data environment

Interviewees shared their experiences with the implementation of nurse‐sensitive data collection, which led to three subthemes each of which provides a summary of the suggested measures to create an enabling environment for data use and uptake in hospitals. The first subtheme covers creating clinician acceptance for data collection; the second covers the establishment of a nurse‐sensitive data culture; and the third focuses on the necessity and benefits of having electronic patient records as a facilitating factor.

##### Creating clinician acceptance for data collection

Several interviewees emphasized the importance of building clinicians' motivation for data collection and analysis. Others shared their experience of clinician's disinterest in data collection (7 out of 18 respondents). In order to build motivation, the interviewees underlined the need to avoid additional workload by data collection and to demonstrate the importance of data:

‘(…) if you put it [the data collection] add‐on to the nurses, then only with an extremely large amount of additional work for the nursing staff and then the Magnet® concept will not find acceptance among the clinicians.’(Interviewee 11)

Having academically qualified nurses (with Bachelor or Master degree) was identified as a facilitating factor to the use and acceptance of data in the hospital and in clinical care. Others mentioned the necessity of building a scientific understanding regarding data collection and analysis among staff (6 out of 18 respondents):

‘And the more academic nurses are on the team, the higher the acceptance that you collect data (…).’ (Interviewee 6)

##### Establishing a data culture

The hospital and teams' attitudes towards data were discussed by 6 out of 18 respondents. On the one hand, it was stated that data are often interpreted negatively, associated with negativity, blaming and pressure to overcome mistakes, missed care or other shortcomings. On the other hand, interviewees suggested a culture change towards a positive approach on using data to overcome this challenge, for example by establishing a positive error culture beyond blaming, based on data and evidence:

‘We are very open and have a culture of making mistakes (…). We don't want to rationalize away the fall, so with us falls are allowed. We just don't want a patient to get a high fall classification, in the worst case get another fracture and has to be operated on again or anything else, but if he falls, that's basically okay, but we want to prevent and avoid a patient getting seriously injured.’ (Interviewee 17)

Three interviewees stated that leaders have an important role when it comes to creating a data culture, by handling data presentation and use in a positive or negative way. The necessity to make data transparent and visible was also mentioned. Examples of in‐house reporting of the data were mentioned, for instance on the wards (paper‐based or digital) or by presenting the data at meetings (8 out of 18 respondents). Interviewees talked about the involvement of clinicians, especially nursing staff, in the selection of nurse‐sensitive indicators or data illustration for internal reporting (5 out of 18 respondents). Additionally, two interviewees mentioned the need to build data literacy by creating new positions and tasks for nursing staff to focus on data collection and monitoring (2 out of 18 respondents). For instance, in one hospital a nurse works half of her time in a typical shift and the remaining time is spent on data and other scientific tasks:

‘So 50 percent typical shift and 50 focused on science. (…) That covers simply this topic, how does the fall rate look, do I have central venous line infections, is everything all right, is it documented correctly. She works on these things continuously.’ (Interviewee 3)

#### 3.2.3 Usage of electronic health records

Hospitals varied in the implementation status of electronic health records as reported by the interviewees, ranging from established electronic health records to hospitals being in the process of implementation (5 out of 18), this was described as having a large impact on data collection. One interviewee described a fully paper‐based documentation. On the one hand, the introduction of electronic health records was associated with high financial and personnel costs, but on the other hand, interviewees suggested that the benefits would outweigh the costs. Electronic health records were suggested to facilitate documentation for and reduce workload of nursing staff. Moreover, interviewees mentioned that electronic health records would enable an automatic and simplified extraction of nurse‐sensitive data (3 out of 18 respondents):

‘So, if you want to expect more from nursing in today's world, where nursing is already at its limit, where the framework conditions are difficult anyway, as far as data evaluation and other things are concerned, then you have to relieve them [nursing staff] on another side. And that only works through a fully digital patient and care record, otherwise it makes no sense.’ (Interviewee 1)

#### 3.2.4 Challenges and opportunities in establishing benchmarking

Material under this theme covered internal benchmarking, the lack of national and European benchmarking as well as, challenges and potential benefits of establishing national and European benchmarking.

##### Implementation of internal benchmarking

Two forms of internal benchmarking were mentioned by the interviewees. In some hospitals, there are inhouse data comparisons between selected wards or departments whereas other hospitals have a hospital‐wide system in place (7 out of 18 respondents). One interviewee described the internal benchmarking structures as follows:

‘(…) we have either just the ward or neurology or the complete department of neurology. We have not done it [benchmarking] outside the neurology center, not even within the (…) hospital (…).’ (Interviewee 6)

In addition, interviewees also mentioned that hospitals belonging to the same hospital group had the advantage of group wide comparisons for nurse‐sensitive data and benchmarking if applied uniformly and consistently (2 out of 18 respondents):

‘That's where we have a practical benchmark from the hospitals in the hospital group.’(Interviewee 3)

Yet, interviewees mentioned that group wide data comparisons existed either for one or few selected nurse‐sensitive indicators or were in the implementation phase.

##### Limited possibilities for national and international benchmarking

In Germany, although there is a benchmarking for one indicator, pressure ulcers, through the mandatory data collection via the German‐wide external quality assurance, its value has been criticized by interviewees. One person mentioned:

‘(…) we have the EQS [external quality assurance] in the field of hospital acquired pressure ulcers, but even there the annual report has (…) quite a high level of aggregation. This has to be closer, (…) more flexible. It should not be based on billing data, in my view. It is [currently] an economic database and not a scientific database (…) Germany still has a lot of work to do to ensure that an adequate benchmark is possible and also brings benefits.’ (Interviewee 18)

Beyond pressure ulcers, interviewees stressed that there is no nationwide benchmarking of nurse‐sensitive indicators for hospitals in Germany. Yet, most interviewees highlighted the necessity of such a benchmarking for Germany to account of the German context (7 out of 18 respondents):

‘It would make sense if we were to benchmark ourselves within Germany, but at the moment we are (…) not yet ready to build up a database.’(Interviewee 17)

In order to establish a nationwide benchmarking, respondents stated structures need to be built, such as an institution, which defines and accepts the data to be compared, and the exclusion of billing data as the data basis (3 out of 18 respondents). Interviewees mentioned benchmarking projects between sub‐groups of hospitals, especially university hospitals (7 out of 18 respondents). Statements ranged from the reporting of development and initial approaches of benchmarking to implemented benchmarking structures across sub‐groups of hospitals:

‘We have [a benchmarking] for falls with some (…) hospitals. [Benchmarking] For pressure ulcer is obvious. But [benchmarking] for catheter associated urinary tract infections and infection rates induced by central venous catheters? No.’ (Interviewee 7)

Due to missing national as well as European benchmarking possibilities (2 out of 18 respondents) some interviewees talked about engaging in benchmarking with the U.S. and related disadvantages due to different country and hospital contexts (3 out of 18 respondents).

##### Motivation for and challenges of setting up benchmarking

The most frequently mentioned benefit of nurse‐sensitive data collection and benchmarking was overall the possibility to improve patient care, mentioned by 10 out of the 18 respondents. One Interviewee said:

‘(…) that you collect data and then also provide evidence‐based care later, yes, and that the patient has a benefit from that’(Interviewee 6)

As interviewees mentioned, nursing quality becomes measurable and comparable through data and benchmarking which should then drive action to improve patient care:

‘(…), where they really find themselves in a competition. So, really looking at the data [of the ward] and question: Why is ward xy always the best and we are …’(Interviewee 16)

Benchmarking would enable competition and trigger an ambition to be better than others, with direct impact on patients' decision to choose a hospital (6 out of 18 respondents):

‘The patients want to be treated where they see a center of excellence. And this is also connected to a Magnet® hospital. You want to be a center of excellence and you want it to stay that way.’ (Interviewee 2)

Also, work performance by nursing staff would become more visible, which could build pride in nurses' performance. One of the interviewees described it as follows:

‘(…) I see this as a remarkable opportunity and also beneficial, that we can use this construct to make excellent care visible and also make it visible in figures, visualize what we do, because nurses accomplish a lot (…).’(Interviewee 18)

Some interviewees suggested that data collection and benchmarking would encourage nursing research, which to date has been limited in Germany:

‘And ultimately, this [data collection on nurse‐sensitive indicators] should also raise questions for research, which should give us clues to ultimately improve patient care.’(Interviewee 3)

Examples mentioned were clinical nursing research, supporting the identification of research questions and clinical practice projects (3 out of 18 respondents).

The interviewees also raised several challenges associated with benchmarking: first, the challenge in identifying suitable comparison groups (6 out of 18 respondents), especially when comparing different departments and specialties with different patient groups.

‘And you can compare them [nurse‐sensitive indicators] with each other. However, the comparison is not very accurate in the sense that there are different specialties. (…) I'll mention ophthalmology now, we have an increasing number of patients with falls, but we don't know whether it's a lot or not. So we are working internally to keep it as low as possible, of course. But the question always comes up, what are they like in other eye clinics? ‘ (Interviewee 5)

Second, the complexity of collecting data on nurse‐sensitive indicators in a standardized way was mentioned as another challenge (7 out of 18 respondents). Interviewees stressed that the definitions of indicators to be collected should be highly standardized and normed. Minimum standards would need to be set up within and across hospitals participating in the benchmark. This was also associated with high costs and staff resources.

‘In the area of data, benchmark, (…) I (…) think that we have to look more into it in Germany or also in Europe, that we have not yet managed to collect and compare data in a uniform way.’(Interviewee 18)

Some interviewees highlighted that a valid benchmarking system requires high data quality and comparability (4 out of 18 respondents).

One example was provided as follows:

‘And often we have also had the experience that we are very good here in collecting data, which is sometimes also to the disadvantage when being compared. Because we record in great detail, and others who do not have such structures as we have, (…) are a little better off on average, because they simply do not have this data [in detail] at all. And then it looks like they don't have these cases [adverse events] either (…).’(Interviewee 5)

## 4 DISCUSSION

This qualitative study shows the limited pre‐existence of nurse‐sensitive indicators in a small number of German pioneer hospitals, striving for Magnet® or Pathway®. Interviewees expressed a high need for developing and implementing additional indicators, and improving data collection. Suggestions were made for creating internal structures for data collection, such as to motivate and involve clinicians, to establish a nurse‐sensitive data culture and to use electronic health records. A need for national and European benchmarking was identified, of which the main benefits of benchmarking were seen in the potential impact on patient outcomes. At the same time, several challenges were identified for setting up a benchmarking system.

### 4.1 Limited pre‐existence of and necessity for nurse‐sensitive data

Many interviewees mentioned pre‐existing data collection for pressure ulcers and falls with injury, yet, levels of implementation varied. The Magnet® designation requires data collection for indicators at the individual ward level and quarterly (American Nurses Credentialing Center, 2021). As for pressure ulcers, although all German hospitals are legally required to collect the data, this is not done at the ward level (Institute for Quality Assurance and Transparency in Healthcare, 2023). Moreover, the quality assurance process has been criticized for limited data validity, an inadequate risk adjustment and the time lag between data collection and reporting (Geraedts & Cruppé, 2022; Geraedts & Selbmann, 2011; Vorbeck et al., 2022). Other nurse‐sensitive indicators, for instance patient fixation or the time between hospital arrival and placing the injection needle when caring for stroke patients (door‐to‐needle time) were existing in some hospitals as mentioned by interviewees but are not routinely collected in Germany. One of the reasons is the lack of political measures or initiatives on data quality and nursing care, monitoring and data collection in hospitals in Germany. In contrast, in long‐term care facilities due to the legal requirement data collection of some nurse‐sensitive indicators is routinely performed (§ 114 b SGB XI).

In the international literature, several additional indicators have been identified as nurse‐sensitive, for instance urinary tract infections, blood stream infections, cancer screening, diabetes care or vein thrombosis, offering a range of possible indicators (American Nurses Credentialing Center, 2021; Burston et al., 2014; Oner et al., 2021; Schreyögg & Milstein, 2016). If these indicators can be transferred to the German context has not been evaluated. It may be influenced by nurses' education, roles and tasks as well as nurses' scopes of practice, which vary across countries (Maier & Aiken, 2016; Maier et al., 2018). In this study, some interviewees suggested to develop their own indicators for their hospitals and specific patient populations. The major rationale was to demonstrate impact tailored to the quality of specific patient populations and the direct contribution of nurses in the process. Additional research is needed to identify indicators for Germany and Europe, develop standardized definitions and data collection. As interviewees stated, collection and comparison of nurse‐sensitive data can improve patients' quality of care. Hence, raising awareness was seen as critical for the uptake of nurse‐sensitive data collection in practice.

### 4.2 Creating an enabling data environment

Interviewees mentioned the need to create an enabling data environment. Several subthemes emerged, including having sufficient nurses with at least Bachelor's degree to foster the acceptance for data collection and use for practice. The RN4CAST study showed that rate of nurses with at least Bachelor degree in Germany was very low in international comparisons (Aiken et al., 2014). In 2012, the German Science Council recommended that 10%–20% of nursing professionals should have at least a Bachelor's degree (German Science and Humanities Council, 2012), with an updated recommendation of 20% in 2023. Yet, despite an increase in Bachelor programmes to educate nurses (German Science and Humanities Council, 2022), and a related reform in 2020 (Federal Ministry of Justice. Gesetz zur Reform der Pflegeberufe (Pflegeberufereformgesetz—PflBRefG), 2017), the academization of nurses in Germany is still in its infancy. Bachelor programmes don't often attract enough students, many programmes do not remunerate students' clinical practice hours, making the Bachelor program financially less attractive than the 3‐year vocational training (German Hospital Federation, 2023). A study analyzing the academisation rate of nursing students showed that in 2021, only 1.75% were enrolled in Bachelor programme at a university or university of applied science, whereas the vast majority were undergoing a 3‐year vocational training (Meng et al., 2022).

In this study, the role of management and leadership was emphasized for establishing a nurse‐sensitive data culture. In the literature, data culture has been defined by four characteristics (Henderson & Powers, 2018). First, sharing of data including transparency, ensuring easy access to the data and fostering understanding of data results. Second, the role of leaders in recognizing and valuing data and for decision‐making. Third, trust in data requiring high data accuracy and validation and fourth, the use of data implying a high relevance for practice (ibid). The interviewees gave several examples with regard to these characteristics, for instance, leaders' critical role in establishing a positive data culture, building data literacy and ensuring data transparency through data visualization for use in clinical care. Education on using and working with data can support building nurses' motivation for data collection and strengthen data literacy.

The advantages of electronic health records for facilitating data collections and reducing workloads were mentioned by several interviewees. A comparison with other European countries showed that German hospitals have on average a low digitalization rate (Stephani et al., 2019). In addition, barriers such as missing interoperability or location requirements (e.g. bandwidth of internet) impede the digitalization (ibid). A legal investment program for hospitals was set up in 2021 to foster digitalization, covering for instance the implementation costs of electronic patient records (Federal Ministry of Health, 2022). Yet, the results of the evaluation of the program are pending.

### 4.3 Challenges and opportunities in establishing benchmarking

Interviewees reported missing national and European benchmarking of nurse‐sensitive indicators and the potential benefits as to improving quality of nursing care. Benchmarking is a requirement by Magnet® (American Nurses Credentialing Center, 2021). Against this background, a group of German hospitals has started an initiative in 2021 to develop a benchmarking system for nurse‐sensitive indicators in collaboration with the German institute for quality and patient safety (BQS institute). For inpatient units, four nurse‐sensitive indicators were selected and defined: pressure ulcers, falls, device‐associated pressure ulcers and multi‐resistant infection rates. For outpatient units two nurse‐sensitive indicators were selected and defined: falls and burns from surgery. Indicators were defined so that on the one hand, the Magnet® requirements were met, and on the other hand, the relevance for the German context was ensured. For example, there are two key figures for the indicator fall; according to the Magnet® definition, falls with injury are to be collected; however, falls that do not result in injury were also considered important by the German hospitals and are therefore also collected, resulting in a second indicator. Data to be collected is not based on billing data, which was also considered important by interviewees in our study (Workgroup, 2022). Billing data are not compiled for scientific purposes and show limitations such as misalignment of incentives in data documentation (Slagman et al., 2023). The challenge mentioned by interviewees of missing or inappropriate comparator groups was also discussed by the benchmark initiative and has been addressed by comparing the results not only at ward‐level but also for specialized departments. Data standardization includes definitions of each indicator and data validation requirements (Workgroup, 2022). Exchange rounds are planned among the participating hospitals to discuss data results and learn from each other to improve the quality of nursing care (ibid). In response to the lack of European benchmarking, the German initiative could be expanded to other European hospitals (ibid), yet this would require a careful assessment of indicators, standardization, quality measures to take care of the various national contexts within Europe.

Whether benchmarking should be mandatory or voluntary, has been an issue of discussion. An assessment of the benchmarking projects funded by the German Federal Ministry of Health suggested voluntary participation in benchmarking projects (Geraedts & Selbmann, 2011). Advantages of voluntary benchmarking were found to contain greater potential for quality improvement in contrast to mandatory benchmarking. Weaknesses of voluntary benchmarking projects may be the small number of participating hospitals and a potential selection bias. Policy measures can support introducing standardized data collections and comparisons of nurse‐sensitive indicators across hospitals by the definition of a core set of indicators.

### 4.4 Limitations and recommendations for future research

The study faces the following limitations. It is an explorative study, which included interviewees from five hospitals implementing Magnet®/Pathway®, being the first ones in Germany undergoing this process. Consequently, the results cannot be generalized.

Future research should focus on the analyzed research question in a larger sample. The Magnet4Europe Study involved over 60 hospitals across Europe and started a large‐scale initiative which could be used to further explore the feasibility of setting up a benchmarking across Europe, including Germany. Furthermore, research is needed to identify nurse‐sensitive indicators for the German and European context. This is an essential precondition to establish standardized nurse‐sensitive data collection across hospitals. As our findings show, nurses' acceptance of data collection and use in practice is important. Further research could analyze measures to enhance nurses' understanding for and work with nurse‐sensitive data in practice.

## 5 CONCLUSION

Due to the limited pre‐existence of nurse‐sensitive indicators in German hospitals and their importance for improving patients' quality of care, there is a necessity for development of indicators and uptake in clinical practice. Further research is needed, to identify relevant nurse‐sensitive indicators for the German and European context.

Hospitals identified conducive internal structures to facilitate data collection, including building acceptance of clinicians, establishing a nurse‐sensitive data culture and the usage of electronic health records. Educational measures can support building nurses' motivation for data collection and strengthen their data literacy. Further research is needed to identify suitable approaches to enhance nurses' data literacy and use in practice through education.

A need for national and European benchmarking of nurse‐sensitive indicators was identified. Policy measures could support the implementation of a standardized data collection of nurse‐sensitive indicators across hospitals.

## AUTHOR CONTRIBUTIONS

Made substantial contributions to conception and design, or acquisition of data, or analysis and interpretation of data; CM, CG, JK, JoK, LA. Involved in drafting the manuscript or revising it critically for important intellectual content; CM, CG, JK, JoK, LA. Given final approval of the version to be published. Each author should have participated sufficiently in the work to take public responsibility for appropriate portions of the content; CM, CG, JK, JoK, LA agreed to be accountable for all aspects of the work in ensuring that questions related to the accuracy or integrity of any part of the work are appropriately investigated and resolved.

## FUNDING INFORMATION

The study was funded by the B. Braun Foundation (CBM, No. 18001021). The funder had no role in the study design, data collection, analysis, decision to publish, or preparation and write‐up of the manuscript.

## CONFLICT OF INTEREST STATEMENT

Claudia B. Maier, Julia Köppen, Joan Kleine and Linda H. Aiken declare to have no conflicts of interest. Carolin Gurisch is working for the BQS institute and part of the project team of the German initiative of benchmarking for nurse‐sensitive indicators. The initiative is briefly described in the discussion section.

## PEER REVIEW

The peer review history for this article is available at https://www.webofscience.com/api/gateway/wos/peer‐review/10.1111/jan.16245.

## Supporting information


Data S1.

Data S2.

## Data Availability

The data that support the findings of this study are available upon reasonable request from the corresponding author. The data are not publicly available due to privacy or ethical restrictions.
