# Supplementary material for: Nurse‐sensitive quality and benchmarking in hospitals striving for Magnet® or Pathway® designation: A qualitative study
Source: J Adv Nurs. 2024 May 27;81(9):5484–96. doi: 10.1111/jan.16245 (PMC12371794; doi:10.1111/jan.16245)
Supplement: Supplementary file 1 — Data S1. [file JAN-81-5484-s001.docx]

**Supporting Information – S1 Appendix**

S1 Appendix: Consolidated criteria for reporting qualitative studies (COREQ): 32-item checklist

Developed from Tong A, Sainsbury P, Craig J. Consolidated criteria for reporting qualitative research (COREQ): a 32-item checklist for interviews and focus groups. International Journal for Quality in Health Care. 2007. Volume 19, Number 6: pp. 349 – 357.

| **No.** | **Item** | **Guide questions/description** | **Notes** | **Page number in manuscript** |
| --- | --- | --- | --- | --- |
| **Domain 1: Research team and reflexivity** | | | |  |
| **Personal Characteristics** | | | |  |
| 1 | Interviewer/facilitator | Which author/s conducted the interview or focus group? | Four interviews were conducted by Claudia Maier, eleven by Julia Köppen and three by Joan Kleine. | Title page |
| 2 | Credentials | What were the researcher’s credentials? E.g. PhD, MD | Dr. PH. Claudia Maier  M.Sc. Carolin Gurisch  M.Sc. Julia Köppen  M.Sc. Joan Kleine  PhD Linda H. Aiken | - |
| 3 | Occupation | What was their occupation at the time of the study? | Claudia Maier was a postdoctoral researcher. Carolin Gurisch, Julia Köppen and Joan Kleine were research associates and doctoral students.  Linda H. Aiken is a professor for nursing science and sociology and director for the Center for Health Outcomes and Policy Research. | - |
| 4 | Gender | Was the researcher male or female? | All researchers are female. | Title page |
| 5 | Experience and training | What experience or training did the researcher have? | Claudia Maier and Julia Köppen have had academic training in qualitative research designs and over 5 years’ experience with qualitative and mixed methods designs.  Carolin Gurisch and Joan Kleine had academic training and experience of qualitative research as well as two years of work experience in this field.  In addition, Joan Kleine has ten years of work experience as intensive care nurse. Linda H. Aiken has extensive experience in quantitative, qualitative and mixed-methods research, including RCTs. | - |
| **Relationship with participants** | | | |  |
| 6 | Relationship established | Was a relationship established prior to study commencement? | For most interviewees, there was no contact between interviewers and participants prior to the study, except for 4 persons who were known professionally to individual interviewers prior to the study. | p.6-8 |
| 7 | Participant knowledge of the interviewer | What did the participants know about the researcher? e.g. personal goals, reasons for doing the research | Most participants did not know anything about the researchers apart from their role in the research, except for the 4 interviewees which knew the interviewers’ interest in Magnet®/Pathway® and improvements of nursing care. | - |
| 8 | Interviewer characteristics | What characteristics were reported about the interviewer/facilitator? e.g. Bias, assumptions,  reasons and interests in the research topic | In each interview the interviewers presented themselves, explained their position at the university and their role in the research. | p. 8 |
| **Domain 2: Study design** | | | |  |
| **Theoretical framework** | | | |  |
| 9 | Methodological orientation and  Theory | What methodological orientation was stated to underpin the study? e.g. grounded theory,  discourse analysis, ethnography, phenomenology, content analysis | Interviews were analyzed according to the content analysis by Mayring. | p. 7-8 |
| **Participant selection** | | | |  |
| 10 | Sampling | How were participants selected? e.g. purposive, convenience, consecutive, snowball | Participants were selected through purposive sampling. In addition, the snowball method was used to identify suitable interview partners in the hospitals fulfilling the applied criteria. | p. 6 |
| 11 | Method of approach | How were participants approached? e.g. face-to-face, telephone, mail, email | Participants were sent an invitation letter via e-mail. | p. 6 |
| 12 | Sample size | How many participants were in the study? | The sample consisted of 18 participants. | p. 7 |
| 13 | Non-participation | How many people refused to participate or dropped out? Reasons? | None. | p. 6 |
| **Setting** | | | |  |
| 14 | Setting of data collection | Where was the data collected? e.g. home, clinic, workplace | All but one Interview were conducted at the participants´ workplaces. All participants were employed in hospitals. One interview was carried out at the interviewer´s workplace, namely the university of Berlin. | p. 7 |
| 15 | Presence of non-participants | Was anyone else present besides the participants and researchers? | No. | p. 7 |
| 16 | Description of sample | What are the important characteristics of the sample? e.g. demographic data, date | The interviewees were chief nursing officers, managers and nursing staff from five different hospitals. The hospitals were chosen due to their efforts to improve the working environment and quality of care via organization-wide reforms (e.g., by implementing Magnet®). | p. 7 |
| **Data collection** | | | |  |
| 17 | Interview guide | Were questions, prompts, guides provided by the authors? Was it pilot tested? | A semistructured interview guide, which was pilot-tested in advance, was used to conduct the interviews. | p. 7 |
| 18 | Repeat interviews | Were repeat interviews carried out? If yes, how many? | No. | p. 6 |
| 19 | Audio/visual recording | Did the research use audio or visual recording to collect the data? | Audio recording was used to collect data. | p. 7 |
| 20 | Field notes | Were field notes made during and/or after the interview or focus group? | Due to audio recording the interviewers did not make notes during the interviews. Lasting Impressions were noted after the interviews. | - |
| 21 | Duration | What was the duration of the interviews or focus group? | Interviews lasted between 30 and 135 minutes. | p. 7 |
| 22 | Data saturation | Was data saturation discussed? | Data saturation was discussed and was relevant to determine the sample size. | p. 7 |
| 23 | Transcripts returned | Were transcripts returned to participants for comment and/or correction? | No. | p. 7 |
| **Domain 3: analysis and findings** | | | |  |
| **Data analysis** | | | |  |
| 24 | Number of data coders | How many data coders coded the data? | Deductive coding was done by Claudia Maier, Julia Köppen and Joan Kleine. Inductive coding was done by Carolin Gurisch. | p. 7-8 |
| 25 | Description of the coding tree | Did authors provide a description of the coding tree? | The complete coding tree is provided in the appendix and also shown in the results. | p. 10 and  supplementary file |
| 26 | Derivation of themes | Were themes identiﬁed in advance or derived from the data? | The topic data collection and benchmarking was identified in advance and coherently included in the interview guide. The identified themes regarding data collection and benchmarking were derived from the data. | p. 7-8 |
| 27 | Software | What software, if applicable, was used to manage the data? | Atlas.ti | p. 7 |
| 28 | Participant checking | Did participants provide feedback on the findings? | No. Transcripts were not given to participants. | p. 7 |
| **Reporting** | | | |  |
| 29 | Quotations presented | Were participant quotations presented to illustrate the themes/ﬁndings? Was each quotation identiﬁed? e.g. participant number | Quotations are used for presenting the results of the study. Each quotation is marked with a participant number. | p. 11-19 |
| 30 | Data and ﬁndings consistent | Was there consistency between the data presented and the ﬁndings? | Yes. | p. 11-19 |
| 31 | Clarity of major themes | Were major themes clearly presented in the ﬁndings? | Three major themes emerged from the interviews, which are named and were used for structuring the results of the interviews. Sub-themes are also named and were used for subheadings. | p. 11-19 |
| 32 | Clarity of minor themes | Is there a description of diverse cases or discussion of minor themes? | The content of the interviews was presented per theme/sub-theme, including the description of diverse cases. Minor themes with no relevance for the research question were summarized under the sub-theme “others” and not reported in the present text. | p. 11-19 |
