# Supplementary material for: Nurse‐sensitive quality and benchmarking in hospitals striving for Magnet® or Pathway® designation: A qualitative study
Source: J Adv Nurs. 2024 May 27;81(9):5484–96. doi: 10.1111/jan.16245 (PMC12371794; doi:10.1111/jan.16245)
Supplement: Supplementary file 2 — Data S2. [file JAN-81-5484-s002.docx]

**Supporting Information – S2 Appendix**

S2 Appendix: Themes, Sub-themes, Exemplary quotes, Extracted codes for data collection and benchmarking

| **Themes** | **Sub-themes** | **Exemplary quotes** | **Extracted codes** |
| --- | --- | --- | --- |
| Limited pre-existence of and necessity for nurse-sensitive data | Pre-existing nurse-sensitive indicators and data collection | “(…) falls with injury, pressure ulcer, much of this has already been measured.” (Interviewee 1)  “What we've already taken on, for example, are these data collections on falls and pressure ulcers for the individual wards." (Interviewee 6)  “Then we said, what is important for us? And then we said, central venous line infection. (…) we need central venous lines in practice. (…). They weren't really captured at all, just imagine.” (Interviewee 3)  "Other areas are starting to collect data for urinary tract infections. We don't have that in my area. (...). We are dealing with the indicators for falls and pressure ulcers. That's what we're working on." (Interviewee 5) | Data collection of pressure ulcers and falls with injury;  implementation status of data collection for further nurse-sensitive indicators |
|  | Inhouse-development of nurse-sensitive data collection | “What is complimentary in our case, (…) is the data collection of quality of life and autonomy indicators. (…) because the challenge is that with these nurse-sensitive indicators, (...) we want to reach a completely different level than is actually typical in the USA. (...) Because a patient does not come to the hospital in order not to get a hospital acquired pressure ulcer or not to get an infection, but to be independent again afterwards, as self-sufficient as possible. That's what nursing is all about, and we wanted to record that in a questionnaire so that the difference between good and not-so-good nursing care could be measured." (Interviewee 1) | Inhouse development of nurse-sensitive data collections |
| Creating an enabling data environment | Creating clinician acceptance for data collection | “(...) if you put it [the data collection] add-on to the nurses, then only with an extremely large amount of additional work for the nursing staff and then the Magnet® concept will not find acceptance among the clinicians." (Interviewee 11)  "And the more academic nurses are on the team, the higher the acceptance that you collect data (...)." (Interviewee 6) | Education level and academization of nursing;  motivation of clinicians |
|  | Establishing a data culture | “So 50 percent typical shift and 50 focused on science. (...) That covers simply this topic, how does the fall rate look, do I have central venous line infections, is everything all right, is it documented correctly. She works on these things continuously.” (Interviewee 3)  "We are very open and have a culture of making mistakes (...). We don't want to rationalize away the fall, so with us falls are allowed. We just don't want a patient to get a high fall classification, in the worst case get another fracture and have to be operated on again or anything else, but if he falls, that's basically okay, but we want to prevent and avoid a patient getting seriously injured." (Interviewee 17) | Content-related involvement of clinicians in data collection and selection;  setup of data literacy for nursing for data collection;  Attitude towards data and facts;  data-based leadership;  data visualization |
|  | Usage of electronic health records | “So, if you want to expect more from nursing in today's world, where nursing is already at its limit, where the framework conditions are difficult anyway, as far as data evaluation and other things are concerned, then you have to relieve them [nursing staff] on another side. And that only works through a fully digital patient and care record, otherwise it makes no sense." (Interviewee 1) | Implementation status of electronic patient record;  Financial and personnel expenses for the implementation of the electronic patient record;  reduction in workload through the electronic patient record |
| Challenges and opportunities in establishing benchmarking | Implementation of internal benchmarking | “(…) we have either just the ward or neurology or the complete department of neurology. We have not done it [benchmarking] outside the neurology center, not even within the (…) hospital (…).” (Interviewee 6)  “That's where we have a practical benchmark from the hospitals in the hospital group.” (Interviewee 3) | Benchmarking within a hospital;  benchmarking within hospital groups |
|  | Limited possibilities for national and international benchmarking | “(...) we have the EQS [external quality assurance] in the field of hospital acquired pressure ulcers, but even there the annual report has (...) quite a high level of aggregation. This has to be closer, (...) more flexible. It should not be based on billing data, in my view. It is [currently] an economic database and not a scientific database (...) Germany still has a lot of work to do to ensure that an adequate benchmark is possible and also brings benefits.” (Interviewee 18)  "It would make sense if we were to benchmark ourselves within Germany, but at the moment we are (...) not yet ready to build up a database." (Interviewee 17)  “We have [a benchmarking] for falls with some (…) hospitals. [Benchmarking] For pressure ulcer is obvious. But [Benchmarking] for catheter associated urinary tract infections and infection rates induced by central venous catheters? No.” (Interviewee 7 ) | External benchmarking – Germany;  external benchmarking – USA;  external benchmarking – Europe;  external benchmarking – groups;  external benchmarking – setup |
|  | Motivation for and challenges of setting up benchmarking | “(…) that you collect data and then also provide evidence-based care later, yes, and that the patient has a benefit from that”. (Interviewee 6)  “(…), where they really find themselves in a competition. So, really looking at the data [of the ward] and question: Why is ward xy always the best and we are …” (Interviewee 16 )  “The patients want to be treated where they see a center of excellence. And this is also connected to a Magnet® hospital. You want to be a center of excellence and you want it to stay that way.” (Interviewee 2)  “(...) I see this as a remarkable opportunity and also beneficial, that we can use this construct to make excellent care visible and also make it visible in figures, visualize what we do, because nurses accomplish a lot (...).” (Interviewee 18)  "And ultimately, this [data collection on nurse-sensitive indicators] should also raise questions for research, which should give us clues to ultimately improve patient care.” (Interviewee 3)  "And you can compare them [nurse-sensitive indicators] with each other. However, the comparison is not very accurate in the sense that there are different specialties. (…) I'll mention ophthalmology now, we have an increasing number of patients with falls, but we don't know whether it's a lot or not. So we are working internally to keep it as low as possible, of course. But the question always comes up, what are they like in other eye clinics?“ (Interviewee 5)  “In the area of data, benchmark, (…), I (…) think that we have to look more into it in Germany or also in Europe, that we have not yet managed to collect and compare data in a uniform way.” (Interviewee 18)  “And often we have also had the experience that we are very good here in collecting data, which is sometimes also to the disadvantage when being compared. Because we record in great detail, and others who do not have such structures as we have, (…) are a little better off on average, because they simply do not have this data [in detail] at all. And then it looks like they don't have these cases [adverse events] either (...)." (Interviewee 5) | Improvement of patient care;  improved visibility of nursing performance;  fostering nursing science;  identifying comparison groups;  data standardization;  data quality |
